# Geographical Mobility, Income, Life Satisfaction and Family Size Preferences: An Empirical Study on Rural Households in Shaanxi and Henan Provinces in China

**DOI:** 10.1007/s11205-015-1102-7

**Published:** 2015-10-12

**Authors:** Jiangsheng Chen, Hong Yang

**Affiliations:** 1 Department of Rural Planning and Resource Management, Northwest A&F University, Yangling, 712100 Shaanxi People’s Republic of China; 2 Department of Foreign Languages, Northwest A&F University, Yangling, 712100 Shaanxi People’s Republic of China

**Keywords:** Family size preference, Rural household, Population, Environment, China, Asia

## Abstract

Employing data from the China rural–urban mobility survey conducted in 2010, this study investigates the influence of family demographic characteristics on the income, life satisfaction, and potential for rural–urban mobility at the rural household level of two provinces of China: Shaanxi and Henan. A larger labor force in a rural household was found to reduce a family’s ability or inclination to move to a city. The findings reveal that family size negatively affects the average income per family member and reduces the marginal income of the labor force and that minor children can improve the life satisfaction of family members. We conclude that a larger family size does not translate to more benefits for a rural household. Family size preference is found to be a reflection of parents’ concerns about elderly care and is deemed to be unfavorable for urbanization in P. R. China.

## Introduction

Recent social, economic, and environmental problems are believed to be primarily related to rapid increases in population (Biddlecom et al. 2005; Potts 2007). Family planning is considered a development tool at the individual and global levels; it allows individuals to gain more control over their lives, ensures a greater balance between population increases and preservation of natural resources upon which some rural families are dependent, and helps improve the global economy (Johnson 1994; Gaffikin 2007). With China being the world’s most populous country, population pressure has captured the attention of scientists and the Chinese government. Since the 1970s, the country has pursued what is termed China’s Family Planning Policy (CFPP), which promotes a one-child-per-couple policy, late marriage, and the postponement of childbirth, advocating that these policies will result in conceiving and rearing healthier children. Efforts are targeted at controlling population growth, improving population quality, increasing household welfare, and alleviating social, economic, and environmental problems. In 2002, the Population and Family Planning Law touted CFPP as a basic national policy and citizens’ duty (Zhai and Gao 2010). Under this policy, only people belonging to small and endangered ethnic groups in remote regions were allowed to have two or more children, but they were not provided with welfare support for these children (Cao and Wang 2010). CFPP has remarkably controlled population, as indicated by the substantial decline in fertility levels and the reduction of average family size (Wang 2009). Perceived environmental and social risks of overpopulation have prompted the Chinese government to not only reduce fertility but also change urban families’ attitudes toward children, influencing the latter’s preference for a smaller family (Larsen 1990). China is believed to have completed the transition to low fertility, but many couples still have strong cultural beliefs that children increase the income of parents (Margolis and Myrskyla 2011), they still prefer having more children, especially in rural areas, where the notion of a distinguished family with several generations living together is pursued, and families with only one child are rare. In rural China, births are underreported, and many families have not adhered to CFPP, raising more than two children (Merli and Raftery 2000). The situation contradicts the government’s notion of resolving China’s issues concerning agriculture, the countryside, and farmers through rural population control.

CFPP was indeed a controversial social decision that sparked discussions among many scholars in China and the rest of the world. Fong (2002) argued that urban daughters have benefited from China’s one-child policy (as stated in the CFPP) and have become more capable of supporting their parents in old age. Attane (2002), Zhang and Goza (2006) assessed the impact of CFPP on reproductive behavior and discussed potential demographic problems such as aging. Disagreement over CFPP has long persisted in academic circles, with some scholars believing that CFPP should be continued in China. For example, Wu (2006) argued that Chinese citizens should be vigilant regarding the country’s population. Yang and Meng (2007) proposed that a stabilized, low fertility level is a precondition for the construction of a harmonious population. Conversely, some scholars have not entirely approved of CFFP. Chen (2005) suggested revising the CFPP to permit bearing a second child and increasing the birth rate in China, considering that population growth is now under control. He likewise noted that the termination of the one-child policy was inevitable in avoiding the risks of having one-child families (Chen 2010). Yi (2008) claimed that CFPP has shaped an inverted pyramid population structure, and that the government should abolish CFPP to improve population quality.

China’s current population is approximately 1.3, 0.713 billion of whom are registered as rural residents (National Bureau of Statistics of China 2012). Hence, rural population development continues to face severe challenges (He 2010). Because CFPP is not very popular among rural families, population and fertility rates should be reduced further in rural China. Urbanization is clearly a part of the development process (Liddle and Lung 2010), and rural-city mobility is viewed as a means to reduce rural population and resolve China’s issues concerning agriculture, the countryside, and farmers. This study does not propose focusing on rural–urban mobility alone because this equates to draining a pool but neglecting the stream pouring into the pool. On the one hand, we recommend facilitating the movement of rural households toward the urban; on the other hand, we advocate reducing the fertility rate or family size in rural areas.

Chinese society has stressed the vital role played by children in the lives of their elderly parents (Zhang and Liu 2007), particularly adult children who are considered important sources of emotional, physical, and financial support for the latter (Shi 1994; Zhang and Liu 2007). Chinese culture encourages having more children, as conveyed in the popular adage, “more children (sons) mean more happiness for a traditional family in China.” Are larger families easier to urbanize? Will having more children bring more happiness? Several studies have focused on the association between well-being and family size (fertility) among Chinese families, but these studies have not employed econometric methods. Moreover, no study has tested the association between rural–urban mobility and family size preference. The primary goal of this study is to analyze the arguments by examining whether larger families are easier to move toward urbanization permanently and whether having more children provides increased benefits to rural families in China and by explaining rural families’ preference for more children. Additionally, this study aims to discover alternative means for controlling rural population besides CFPP because no society has lowered its birth rate using a single method of family planning (Potts 2007).

This study presents two main developments. The first pertains to the sample used in this study, which includes households that have resided in urban areas for the past 3 years and rural families. The data and the results will help in gaining a comprehensive understanding of the migration of rural households. The second development explores the possibility of rural–urban mobility when assessing family benefits. Given the major changes in socio-economic structure generated by China’s economic institutional reform since 1978, living and working conditions have improved more in urban than in rural areas, and the number of surplus agricultural laborers migrating to cities and towns has increased. We believe that the movement of rural households to an urban area or town benefits such families and represents a development process for a country.

The rest of this study is organized as follows. Section 2 provides the theoretical framework. The data source is introduced in Sect. 3, and the estimation results are presented in Sect. 4. Section 5 offers conclusions and suggestions.

## Theoretical Framework

The objectives of this study are to examine the effects of family size preference on the possibility of rural–urban mobility among rural families, satisfaction with life and income in rural families in China and to explore the reasons behind rural family size preferences. The results of this study should contribute to the discovery of ways to decrease the rural population and to smooth the urbanization process in China.

This study stresses that the household is the basic unit for conducting policy; families’ decisions to have more children are based on a comparison between the expected returns and costs; and their family size preferences have impacts on benefits at the individual level and urbanize process at the country level.

We would expect, on average, higher levels of benefit to be associated with the following: the possibility of rural–urban mobility, which can impact the urbanization process at the country level, higher family income, and life satisfaction. Thus, this study analyzes real benefit in terms of these three aspects. First, since 1978, an increasing number of families have moved into cities or urban areas. This change can be attributed not only to urban agglomeration economies (Finney and Kohlhase 2008) but also to the gaps between the living and working conditions of rural areas and cities (or urban areas) in China. Given that the quality of life is better in cities than in rural areas, we employ the probability of moving into urban areas to describe the real benefits for rural families. Second, a family’s gross income is a common index for measuring family benefits, but it does not clearly reveal the levels and the ability of each family member to create wealth because population size and structure differ across China’s rural families. In addition to gross family income, the influence of household demographic characteristics on family members’ average incomes and annual income per labor force participant are discussed. These three income indicators comprise the family income evaluation system adopted in this study because these indicators complement each other and measure family income from different perspectives. Third, the tradition of saving and retaining money over long periods of time is common among Chinese families; therefore, an income indicator is insufficient to reveal the true benefits for high-income families. Life satisfaction is frequently used when evaluating subjective benefit. A number of recent studies have focused on life satisfaction in measuring the level of well-being among families (Nielsen et al. 2010; Knight et al. 2009; Ji et al. 2002; Cwikel et al. 2006). Life satisfaction is defined as the overall assessment of an individual’s quality of life according to his/her personal judgment and criteria (Amit 2010). Studies on the relationship between family size (or children) preferences and life satisfaction that have primarily focused on the association between childlessness and psychological benefit have yielded mixed findings. For example, Lowenstein et al. (2007) found that filial norms are negatively related to life satisfaction in older people, whereas Hansen et al. (2009) indicated that childless women report significantly lower life satisfaction compared with mothers with residential children and empty-nest mothers. However, among men, parental status is unrelated to any of the aspects of psychological benefit. We evaluate the relationship between family members’ life satisfaction and family population characteristics in rural China.

The definition of family size is another important aspect that requires explanation. Having more children generally results in a larger population for a traditional rural family in China. This study focuses on family population, and certain issues are addressed. First, gender differences are ignored. Given CFPP and the Chinese preference for sons, we can speculate that the first child in families with more than one child is more likely to be a girl and that the second or the third child is a boy. In one-child families, the child is more likely to be a boy. Second, intergenerational relationships are not considered in discussing children in this study. Traditional Chinese rural families generally have several generations living in one house. Therefore, differentiating a father from a son is not necessary in the dynamic perspective. Third, to reflect family population and its structure, three variables are selected, namely, family size, number of family members in the labor force, and number of minor children; different combinations of these variables can reveal different population preferences and other population characteristics among families.

A logit regression model is employed to evaluate how having an additional member influences the possibility of rural-city mobility in rural families and family members’ life satisfaction. The model is expressed as follows:

1 $${\text{Prob}}(Y_{i}^{j} = 1) = \frac{{e^{{\beta^{\prime } X_{i} }} }}{{1 + e^{{\beta^{\prime } X_{i} }} }},$$

where *Y*^1^ and *Y*^2^ denote two independent models and *j* is coded either as 1 or 2 in these models. The model is used to estimate the influence on the probability of rural-city mobility of a rural family when j is 1, and the dependent variable *Y*^1^ takes a value of 1 when the family has resided in an urban area; otherwise, *Y*^1^ is 0. The model likewise estimates the influence on family members’ life satisfaction when *j* is 2, with *Y*^2^ as the dependent variable of satisfaction. When family members feel satisfied, i.e., their expected benefit exceeds costs, then *Y*^2^ takes a value of 1; otherwise, *Y*^2^ is 0.

Equation (1) can be transformed into the following equation:

2 $$L_{i}^{j} = \ln \left( {\frac{{P_{i}^{j} }}{{1 - P_{i}^{j} }}} \right) = \beta^{\prime } X_{i} .$$

Generally, Eq. (2) is termed the linear expression of the parameters. A linear equation facilitates the explanation of estimated parameters. A sample expression of *β*_1_ is provided in Eq. (3),

3 $$\frac{\partial z}{{\partial x_{1} }} = \beta_{1} ,$$

where $z = \ln \frac{{P_{i}^{j} }}{{1 - P_{i}^{j} }}$. By taking the antilogarithm of the estimated coefficient (*β*_1_), we obtain the odds ratio, or the change in the ratio of the probability of a particular event (*Y* = 1) to the probability of a reference event (*Y* = 0) for a unit increase in an independent variable (*x*_1_) while holding other variables constant. This study estimates Eq. (2), then explains parameters using Eq. (3). Maximum likelihood estimation is employed to calculate the parameters for the binary logit model using SAS 9.2 software.

A linear equation is employed to study the influence of family size and structure on a rural household’s income, including annual family income, annual average income of each family member, and annual average income of labor force participants. The model is expressed as follows:

4 $$\ln Y_{i}^{j} = \alpha + \beta^{\prime } X_{i} + u_{i} .$$

The superscript *j* is used to designate the independent models *j* = 1, 2, 3, respectively. The regressands *Y*^1^, *Y*^2^, and *Y*^3^ represent annual family income, average income of each family member, and annual income per labor force participant, respectively. These three variables are used in their logarithmic forms in the regression analysis. The vector *X* is a column of factors that influence family income, including variables regarding the vocation of labor force participants, local regional information, and those expressing family population characteristics, which is a main theme of this study. The parameter *β* is a column vector, whereas *u* is the residual error for *i* = 1,…,*n* individuals. This study adopts an ordinary least squares (OLS) method in estimating the parameters using SAS 9.2 software.

Multicollinearity is a common issue, especially in cross-sectional data; although it is simple to diagnose, no satisfactory solutions for this issue are currently available. Principal component analysis and elimination of variables with low tolerances are commonly used methods (Allison 1999). This study eliminates variables with tolerances that are lower than 0.71 based on the multicollinearity diagnosis.

## Data Source and Description

Conceptual models of decision-making in related literature tend to view households as unified social units, and empirical tests of these models often regard the views of one person as the view of the entire household (Coulter et al. 2012). Similarly, this study is based on a rational family hypothesis that considers livelihood pattern as the decision of all family members in pursuit of utility maximization for the whole family. Therefore, we treat the family as the research unit, wherein every family member understands the family’s decisions and holds a similar characterization of their family situation and life; hence, the respondent can always provide accurate information in a survey.

Data from the China Rural-City Mobility Questionnaire Survey (CMQS) conducted in August 2010 was employed in this study. The questionnaire consisted of items gathering data on family characteristics and vocational information: population, average age and educational level of the main labor force participants, family income, and any change in residence comprised the former, whereas information regarding changes in workplace, departments, and so on comprised the latter. Respondents provided family background information, whereas regional information consisting of figures issued by local statistics departments was obtained by researchers based on the locations provided.

This survey was conducted among 4000 rural households and 4000 urban households (one questionnaire for one household), with response rates of 67 % (2680/4000) and 59.2 % (2368/4000), respectively. For the survey is conducted at random and only 138 urban households are new ones from rural areas in 3 recent years, we extracted these 138 questionnaires from 2368 urban questionnaires. Unlike in related literatures, this study combines 2680 questionnaires of rural families with 138 questionnaires from urban families (a total number of questionnaire is 2818 for this study). Data consistency was checked by comparing the household income; some observations with extreme income were excluded, and those with missing values also were deleted. The final sample size was 1552. The samples not only met the criteria for a large sample but also covered the 30 counties in Shaanxi and Henan Provinces in China. The definitions and statistical descriptions of the variables considered are presented in Table 1, the average values of variables are similar to the summary statistics reported in China Statistics Yearbook (2012); the results revealed that the standard deviations of variables are concentrative. We determine that the statistics of the samples can reflect Chinese reality and general information on Chinese rural population.

**Table 1:** Definitions and statistical descriptions of related variables

Variable	Definition	Mean	SD
inco	Annual family income (10 thousand RMB)	2.208	1.521
mpin	Average income of family member (10 thousand RMB)	0.540	0.407
mfin	Annual income per labor force (10 thousand RMB)	1.073	0.852
mage	Average age of main labor forces	41.475	5.769
ethn	Ethnicity: code 1 is Han people; otherwise 0	0.935	0.247
educ	Average education of family’s main labor forces (year)	7.953	2.891
pfam	Size of family population	4.381	1.097
yoch	Number of minor children	0.885	0.829
labf	Size of family’s main labor force	2.256	0.876
fapl	The place where the family lives: code 1 is city (urban); otherwise, 0	0.094	0.211
sati	Satisfaction with life: code 1 is satisfied with life; otherwise, 0	0.801	0.399

## Results and Discussions

### The Impact of Rural Family’s Demographic Characteristics on Rural–Urban Mobility

Urbanization theories hold that population concentration can effectively reduce market transaction costs (Finney and Kohlhase 2008) and that urbanization is a result of rural populations pursuing their own interests. In addition to having a relatively higher family income, a city resident can also enjoy the social benefit of using communal facilities; the greater the probability of rural–urban mobility is, the more likely rural families are to enjoy urban benefits.

In developed countries, urbanization is inversely related to family size decline (Guo et al. 2012); however, the nature of this relationship is not well understood at the rural family level in China. We use Eq. (2) to estimate the impact of family size and instruction on the probability of rural–urban mobility for a rural family.

Table 2, which only contains the variables concerning the subject of this study, shows the estimated results of how household characteristics affect the probability of rural–urban mobility. We can find that only the variable Labf has a weak statistic significance at the 10 % level among the core variables (Pfam, Yoch, Labf), and the estimated parameter is −0.3232, suggesting that the population characteristics in a rural household don’t have an effect on the probability of rural–urban mobility, or have a weak effect suggesting that the probability of rural–urban mobility will decrease when the number of laborers in a rural family increases. Chen and Zheng (2005) suggested that the preference for local work of certain farmers is based on rational decisions instead of traditional sentiments for their hometown. This study agrees with said argument and proposes two reasons why a family composed of more laborers may choose not to migrate. First, a rural family may experience difficulty in availing of the benefits offered to urban residents given the stringent limitations in China’s household registration system. Second, families composed of more laborers have sufficient local social capital and produce higher gross earnings, thus preventing them from qualifying for these benefits (But these two reasons that we proposed are speculations need to be empirical tested in further researches.).

**Table 2:** The impact of a rural family’s demographic characteristics on the possibility of rural-city mobility

Variable	Estimate	Odds ratio
Intercept	−4.6114*	
Mage	−0.0127	0.987
Ethn	−0.1914	0.826
Educ	0.1169***	1.124
Pfam	−0.1638	0.849
Yoch	−0.00634	0.994
Labf	−0.3232*	0.724
Inco	0.3397***	1.405
Likelihood ratio (Pr > ChiSq)	<0.0001	
Sampling size	1437	

*** 0.01; ** 0.05; * 0.10

The parameter for the *yoch* variable is statistically insignificant, suggesting that the number of minor children has no significant influence on the probability of rural–urban mobility. Some scholars consider children’s education as a factor for urbanization, which is worthy of further examination; in the abovementioned results, children’s education may not have sufficiently driven the rural families to move to urban areas. Moreover, family size, which is in fact negative, has no significant effects on migration ability.

We can also find that two non-core variables, Educ and Inco, are statistically significant at 1 % level, their parameters are 0.1169 and 0.3397 respectively, these are interesting results, and seem to suggest that: (1) rural families with better educated economically active members have a greater likelihood of rural–urban mobility; and (2) rural families with greater financial resources are more inclined to migrate to urban areas. This is possibly because they have greater prospects of getting paid employment.

### The Impact of Rural Families’ Demographic Characteristics on Family Income

This study uses Eq. (4) to estimate the separate influences of family income, average income of each family member, and average annual income of main labor force participants. The results are shown in Table 3 (which only lists variables concerning the subject of this study), in which each column describes an estimated result and all three regressands have been transformed by the natural logarithm.

**Table 3:** The impact of a rural family’s demographic characteristics on family income

Variables	Total income	Average income per family member	Average income per labor force
Intercept	1.026	0.704	1.336
	(2.27)**	−1.55	(2.90)***
mage	−0.044	−0.0518	−0.0532
	(−2.03)**	(−2.37)**	(−2.40)**
mage^2^	0.000556	0.000640	0.00068273
	(2.08)**	(2.38)**	(2.50)**
ethn	−0.00726	−0.0209	0.00874
	(−0.11)	(−0.31)	(−0.13)
educ	0.02359	0.0244	0.0232
	(3.94)***	(4.07)***	(3.81)***
pfam	−0.079	−0.295	−0.0621
	(−4.49)***	(−16.71)***	(−3.46)***
yoch	0.041	0.036	0.0226
	(1.91)*	(1.68)*	(−1.03)
labf	0.201	0.202	−0.224
	(8.96)***	(8.94)***	(−9.80)***
R^2^	0.210	0.324	0.227
Sampling size	1403	1403	1403

Total income refers to total annual family income; Average income per labor force refers to average annual income per labor force

t-statistics are given in parentheses

*** 0.01; ** 0.05; * 0.10

The results indicate that increasing the number of laborers in a family can efficiently improve family income. Model 1 (shown in Column 2) suggests that the number of labor force participants in a family is statistically significant and that family income increases by 20 % when another member of the family joins the labor force, holding other variables constant. In a general case of a family with two workers, family income will increase by 50 % (We can suppose that every labor has a same income per year—that is 100 units. For a family with two workers, the total income is 200 units a year; for a family with three workers, the total income is 300 units a year and the increased income is 100 units due to the increased labor. The three workers family increased the income by 50 %, compared with two workers family.) when another member joins the labor force, holding income per worker constant; however, the marginal income for this additional worker is lower in this model at approximately 20 %. Based on the results, having more than five laborers is considered ideal for a rural family. Model 3 (Column 4 in Table 3) suggests that the annual income per laborer will decrease if the number of laborers grows, which is the microeconomic embodiment of the involution of agricultural labor inputs (Feng and Ren 2008).

Only two of the parameters for the variable yoch are weakly significant at the 10 % level, we can think that the number of minor children has not or has low possibility to influence on family income. If it has, the number of minor children is expected to positively influence family income and average income per family member, both parameters approximately measuring 4 %, which is relatively low. A possible explanation is that the consumption of minor children is lower than that of the elderly, or that minor children can participate in family work as semi-skilled laborers (He and Dong 2009).

Different combinations of the three family demographic indicators reveal different rural family structures. For example, keeping the family population and labor force size constant, the family is considered a young family when the number of minor children increases; otherwise, it is an old family. When the number of minor children and the number of laborers remain constant, increasing the family size indicates that there are more elderly, non-working members in a given family. Estimation results show that the parameters for the variable *pfam* are significantly negative in the three models, which indicates that increasing the number of non-working elderly members will reduce family income, average income per family member, and laborers’ average incomes. Thus, workers not only support the elderly financially but also provide more effort in caring for them. This finding proves the revenue utility (Li 2004) and insurance utility (Li 2004; Jiang 2010) of adult children in rural families. Rural families are not entitled to receive insurance from the government or from society in rural China. Hence, older people have to depend on their adult children for support; insurance utility can be considered a reason for rural families’ preference for more children or a larger family in this case.

In reviewing Sect. 4.1, we infer that rural households’ preference for housing more generations and workers is a logical reaction to the lack of a rural insurance system.

Variables, mage and mage^2^, are both significant at the 5 % level, and we can find by calculation that the dependent variables will be largest in three models when the average age of main labor forces is about 40, suggesting that keeping the average age of main labour forces in a rural family be 40 years old can make a rural family get more total income, average income per family member and average income per labor force, and that may be the reason why giving birth to a baby earlier is a preference for most rural families in China.

The parameters of variable educ in three models are all significant at the 1 % level and they are 0.235, 0.244 and 0.232 respectively. These parameters suggest that family income will improve about 2.4 % if the average education year of family’s main labor forces increases one year.

### The Impact of Rural Family’s Demographic Characteristics on Satisfaction with Life

Considering the universality of utility maximization, rural families pursue both pecuniary income and non-pecuniary effects, and life satisfaction is used in this study to measure their overall benefits. Although computing non-pecuniary benefits and costs is difficult, family members are deemed subjectively satisfied when their expected returns exceed expected expenditures.

The questionnaire measures satisfaction on a scale of 1–5, with scores representing *quite dissatisfied, relatively dissatisfied, satisfied, relatively satisfied*, and *quite satisfied*, respectively. This study reclassifies these five levels into *dissatisfied* when the value is less than 3 and *satisfied* above this value. The influence of a rural family’s demographic characteristics on family members’ life satisfaction is estimated using Eq. (2), and the results are presented in Table 4. Among the demographic characteristics, none of the core demographic variables was significant at the standard 5 % level, suggesting that the popular saying in China, ‘the more children, the more blessings’, is not necessarily the case, even though the variable yoch, the number of minor children, is significant at the weak level of 0.1 with a positive parameter, family members are lower likely to be satisfied when the number of minor children grows, reflecting the utility effects of children’s consumption. However, this effectiveness fades as children grow older; in other words, a flourishing population (*ren ding xing wang*) cannot ultimately provide satisfaction to rural parents.

**Table 4:** The effect of a rural family’s demographic characteristics on life satisfaction

Variable	Estimate	Odds ratio
Intercept	−1.4266	
Mage	0.00585	1.006
Ethn	0.2715	1.312
Educ	0.00905	1.009
Pfam	−0.0791	0.924
Yoch	0.1591*	1.172
Labf	0.1361	1.146
Likelihood ratio (Pr > ChiSq)	<0.0001	
Sampling size	1435	

*** 0.01; ** 0.05; * 0.10

## Conclusions and Suggestions

The Chinese notion that “more children bring more benefits” is considered old-fashioned. Whereas prior research has focused on the link between income and fertility and the effects of childlessness on the psychological well-being of the elderly, the current paper investigates the effects of having more children (larger families) on family income, life satisfaction, and the probability of urbanization of rural families in China.

In summary, a rural family’s willingness and ability to migrate would decline if the family were to gain more workers. This study posits that rural families with more workers have more social capital, are powerful in the local area, and can easily find work locally. By contrast, a rural family composed of fewer workers must leave the rural area because of limited work opportunities. Compared with families with more workers, families with few working members always fails in competing for local work opportunities. Such families have lower income and experience difficulty in migrating to an urban area permanently; workers in these families must move from rural to urban areas seasonally. Therefore, the mobility of rural families to urban areas is impeded by rural families’ larger size and is regarded as a low-efficiency process in China.

We also find that increasing the size of a family does not effectively increase a rural family income. Even if the number of workers increases, evidence confirms diminishing marginal returns. Population characteristics variables have few effects on the life satisfaction for a rural family, although variable “Yoch” affects the life satisfaction of family members at a significant level of 10 %. As Feng and Ren (2008) noted, fertility is a diseconomy in China’s rural areas, and rearing children is similar to investing in durable consumer goods. Along with discussions on the influence of family income in Part 4(b), this study mainly concludes that more children will not lead to an improved welfare but finds the evidence of supporting the idea of rearing a son for elderly care, and that birth earlier for a couple is better strategy in a rural family, indicating that having more adult children increases the likelihood of an elderly receiving support. Such evidence explains why many families, especially those in rural areas, prefer having more children. The pursuit of more children is a corresponding response to the current structure of rural life, where insurance for aged care is lacking. As another popular Chinese adage preaches, “a son is insurance for old people”—elderly persons living with household members are happier than those living alone (Yang and Meng 2007). However, family size preferences have diminishing marginal effects on increasing family’s income and a negative effect on rural–urban mobility.

China’s government has determined basic strategies for rural–urban integration, realizing the permanent migration of surplus rural labor (Yao et al. 2009). These strategies have been developed to encourage rural families with more members to decrease their fertility or permanently migrate to urban areas. Based on all of the studies cited above, some suggestions are made. First, the rural endowment insurance system requires the active promotion of reforms to convince rural parents that their later livelihood will be guaranteed, thereby eliminating the concept of rearing sons to care for them in old age. We argue that social insurance can effectively replace family insurance because elderly persons living in nursing homes express higher levels of life satisfaction than those living in households mostly with their children (Zhang and Liu 2007). The second suggestion involves removing obstacles to the migration of rural families, thus facilitating their migration and promoting stable lives. Urbanization is a development process; it not only promotes the well-being of rural families but also reduces fertility (Romaniuk 2011). This phenomenon can be explained by the fact that an urban resident is entitled to social insurance for elderly care in China, which replaces the need for family insurance. Third, the construction of town systems in affluent areas with surplus rural laborers must be strengthened to create more local off-farm employment opportunities. Many Chinese cities are overly populated because of economic imbalance among regions, and the present infrastructure will be incapable of coping with the demand (Zang and Li 2002). A balance in economic and employment opportunities among regions is sought; policies should encourage enterprises to locate in small towns or developing regions to allow citizens from less powerful rural families to easily find work, ultimately leading seasonally mobile families to return to their hometowns and achieve permanent migration. On the one hand, big cities would be relieved of population pressures; on the other hand, the economies of smaller towns would be developed, thereby achieving parallel development among big cities and small towns.

A few limitations are noted. Observational studies are rarely based on purely random samples, intentionally or unintentionally (Cameron and Trivedi 2005; Gujarati 2004), but the results of this study are largely dependent on the survey data employed; a deterministic theory can be invalidated by a single contradictory observation (Greene 2003). Further investigation is necessary to validate the accuracy of the results of this research.
